# Assessing aesthetic outcomes of different incision types for nipple-sparing mastectomy followed by radiation therapy in prepectoral direct-to-implant breast reconstruction: a retrospective study

**DOI:** 10.1186/s12957-025-03730-4

**Published:** 2025-03-15

**Authors:** Jae Hoon Jeong, Ha Eun Park, Eun-Kyu Kim, Chan Yeong Heo, Chongsoo Park

**Affiliations:** 1 Department of Plastic and Reconstructive Surgery, Seoul National University Bundang Hospital, Seoul National University College of Medicine, Seongnam, Republic of Korea; 2 Graduate School of Medicine, Konkuk University, Chungju-si, Republic of Korea; 3 Department of Surgery, Seoul National University Bundang Hospital, Seoul National University College of Medicine, Seongnam, Republic of Korea; 4 Department of Plastic and Reconstructive Surgery, Inje University Busan Paik Hospital, Inje University College of Medicine, 75 Bokji-ro, Busanjin-gu, Busan, 47392 Republic of Korea

**Keywords:** Breast, Breast neoplasms, Mastectomy, Breast implants, Surgical wound, Aesthetic outcomes, Post-mastectomy radiotherapy (PMRT), Prepectoral Direct-to-implant (DTI) reconstruction, Nipple symmetry, Seoul breast esthetic scoring tool software (S-BEST), Inframammary fold (IMF) incisions

## Abstract

**Background:**

This study analyzes the aesthetic outcomes associated with inframammary fold (IMF) incisions compared to radial incisions, with or without a periareolar component (referred to as periareolar/radial, PR), considering nipple-sparing mastectomy (NSM) followed by prepectoral direct-to-implant (DTI) reconstruction and subsequent post-mastectomy radiotherapy (PMRT). We assessed changes in breast and nipple symmetry, nipple-to-IMF distance, and nipple Y-axis coefficients to understand how different incisions influence post-radiation aesthetic outcomes.

**Methods:**

Forty patients who underwent NSM and prepectoral DTI reconstruction followed by PMRT between September 2019 and December 2022 in a single institution were included. Patients were divided into PR incision (*n* = 9) and IMF incision (*n* = 31) groups, with the latter further separated into IMF 1 group (surgeries from 2019 to 2021, *n* = 13) and IMF 2 group (surgeries in 2022, *n* = 18). Pre- and postoperative (6–18 months after surgery) analyses of body measurements and medical photographs were conducted using the Seoul Breast Esthetic Scoring Tool (S-BEST) software, developed by same institution, to calculate breast symmetry scores, nipple-to-IMF distance, and nipple Y-axis coefficients. Statistical analyses assessed differences between groups.

**Results:**

All groups showed decreased breast symmetry scores postoperatively (PR group: -1.111, IMF 1 group: -0.539, IMF 2 group: -0.389) and increased nipple-to-IMF distance (PR group: 0–0.2 cm, IMF 1 group: 0.2–0.5 cm, IMF 2 group: 0.3–0.4 cm). The changes in nipple y-axis coefficients were minimal across all groups. And the PR group received a lower average radiation dose (47.64 ± 5.2 Gy) than IMF 1 group (54.45 ± 5.28 Gy) and IMF 2 group (54.07 ± 4.79 Gy). Statistical analysis indicated no significant differences across the groups (*p* > 0.05, Kruskal-Wallis test).

**Conclusions:**

While IMF and PR incisions yielded similar aesthetic outcomes post-radiation, IMF incisions showed trends toward better symmetry, especially at higher radiation doses. These findings support the IMF incision as a favorable choice in NSM with DTI reconstruction followed by PMRT, though patient anatomy and preferences remain critical for surgical planning.

**Supplementary Information:**

The online version contains supplementary material available at 10.1186/s12957-025-03730-4.

## Introduction

Breast cancer management has evolved significantly over the years, with an aim not only on avoiding recurrence rates but also for optimizing quality of life through better aesthetic outcomes [1]. Among modern surgical techniques, nipple-sparing mastectomy (NSM) with immediate reconstruction has allowed patients the opportunity to preserve their breast with a more natural breast contour [2]. For reconstruction options, prepectoral direct-to-implant (DTI) breast reconstruction has gained popularity due to its less invasive nature and the avoidance of muscle manipulation, potentially leading to a quicker recovery and less discomfort postoperatively [3]. However, the aesthetic outcomes of this procedure can be significantly influenced by multiple factors, including the choice of incision type, especially when followed by post mastectomy radiotherapy (PMRT), which can alter tissue quality and impact final outcome [4, 5].

PMRT plays a vital role in reducing cancer recurrence, but often presents challenges for reconstructive surgery due to its effects on skin texture, elasticity, and vascular supply [6]. Radiation induced fibrosis, skin retraction, and tissue contracture can compromise aesthetic outcomes in terms of symmetry, nipple to inframammary fold (IMF) distance, and nipple Y-axis coefficient, by increasing the risk of healing complications and compromising the overall health of the tissue after reconstruction [5]. While the benefits of PMRT in reducing recurrence of breast cancer are well-established, its effects on the aesthetic results of NSM with prepectoral DTI reconstruction remain a subject of ongoing research. Specifically, the influence of different incision types used during NSM on post-radiation aesthetic outcomes is not well understood. Therefore, it is important to determine the aesthetic outcomes of the operation after PMRT.

The IMF and periareolar/radial (PR) incisions represent two widely used approaches in NSM, each with unique benefits and drawbacks [7]. IMF incisions, positioned discreetly beneath the breast, are known for their lower scar visibility and potential to maintain breast contour post-reconstruction [8]. However, there may be cases where the lower pole flap of the breast is dissected thinly and accessing the upper pole area can be difficult. PR incisions, on the other hand, offers easy access to the nipple areolar complex (NAC), but are associated with a higher risk of complications and scarring due to their location on more visible parts of the breast [7]. The incision location not only plays a crucial role in the surgical and oncologic aspects of the procedure but also significantly impacts the cosmetic results and patient satisfaction. Therefore, understanding the implications of different incision types is essential for careful surgical planning to optimize aesthetic results and enhance patient satisfaction following breast reconstruction [9].

Given the importance of balancing oncologic safety with aesthetic and functional outcomes, this study focuses on comparing the aesthetic outcomes of IMF incisions versus PR incisions in NSM followed by prepectoral DTI reconstruction and subsequent adjuvant radiotherapy (RT). We specifically assessed breast and nipple symmetry, changes in the nipple to IMF distance, and nipple positioning before and after radiation therapy, to understand how different incision choices influence post-radiation aesthetic outcomes. By conducting a retrospective analysis of patient outcomes, this research contributes valuable information to the ongoing discussion on optimizing surgical techniques in breast cancer patients. To recap, the aim of this retrospective study is to evaluate the aesthetic outcomes of IMF versus PR incisions in patients undergoing NSM followed by prepectoral DTI breast reconstruction and PMRT.

## Methods

### Design and ethical approval

The retrospective study reviewed records of patients that underwent NSM followed by prepectoral DTI breast reconstruction and PMRT at a single tertiary center from September 2019 to December 2022. Institutional ethical approval (IRB No. B-2312-871-101) was obtained from the Ethics Committee of Seoul National University Bundang Hospital (Seongnam, Republic of Korea).

### Patients

A total of forty patients who underwent NSM and prepectoral DTI reconstruction unilaterally followed by PMRT were included. Inclusion criteria were adult women (age 30 to 65) diagnosed with breast cancer who opted for NSM with prepectoral DTI reconstruction and were scheduled for PMRT (Table 1). Patients with body mass index (BMI) categorized as underweight (< 18.5) or obese (≥ 30) were excluded to avoid potential confounding effects from extreme body weights [10]. Individuals younger than 30 or older than 65 were excluded to ensure the study focused on a typical population for breast surgery and RT. Patients who underwent surgeries using techniques other than periareolar, radial incision, or IMF were also excluded to maintain consistency in surgical methods. Finally, participants lacking postoperative nipple-to-IMF measurements were excluded, as this data was essential for assessing symmetry outcomes.

**Table 1:** Inclusion and exclusion criteria

Inclusion Criteria	Exclusion Criteria
• 30–65 years of age • BMI of 18.5–29.9 (normal to overweight range) • Underwent NSM with PR or IMF incisions followed by PMRT. • Completed RT (16–35 sessions, total dose 42.56–60 Gy) • Documented preoperative and postoperative symmetry score	• Ages outside the specified range • BMI outside the specified range • Underwent alternative surgical techniques. • Did not complete RT • Incomplete medical records

BMI: Body mass index, NSM: Nipple-sparing mastectomy, PR: Periareolar/radial incision, IMF: Inframammary fold incision, PMRT: Post-mastectomy radiotherapy, RT: radiotherapy, Gy: Gray

Patients were divided into two groups based on the type of surgical incision: PR incisions (*n* = 9) and IMF incisions (*n* = 31). The PR incisions group included patients that had surgery performed using radial incision with or without a periareolar incision. The IMF group was further categorized into IMF 1 group (surgeries performed between 2019 and 2021, *n* = 13) and IMF 2 group (surgeries performed in 2022, *n* = 18), reflecting a shift toward IMF at the institute as the preferred technique due to consistently superior postoperative outcomes, such as aesthetic symmetry, scar concealment, and patient satisfaction. This shift reflects an evidence-based evolution in surgical practice, where the choice of technique was refined over time to prioritize optimal patient outcomes. The patients were also divided into subgroups according to cancer stage (0 to 3) and pathology (mucinous carcinoma or MC, invasive lobular carcinoma or ILC, invasive ductal carcinoma or IDC, both ILC/IDC, and ductal carcinoma in situ or DCIS) to further investigate the differences between aesthetic outcomes depending on the cancer stage and pathology.

### Surgical procedure

All surgeries were performed by a team consisting of a specialized breast surgeon (E. K.) and a reconstructive surgeon (C.Y. H.). Unilateral NSM was performed using PR or IMF incisions, followed by immediate prepectoral DTI reconstruction. All patients received an implant wrapped in acellular dermal matrix (ADM). No incisions were created in the ADM to fabricate a mesh configuration; rather, the ADM was employed in its initial form. Patients subsequently underwent PMRT tailored to their cancer stage and pathology.

### PMRT protocols

Radiation oncologists at the institute were involved in defining the target structures [11]. The designated target structures included the chest wall, three tiers of axillary lymph nodes, including Rotter’s nodes, and the internal mammary nodes (IMN) and supraclavicular region (SCV) when indicated. The regional nodes have also been included in some cases. The chest wall encompasses all soft tissue located anterior to the pectoral, costal, and intercostal muscles, extending 3 mm below the skin’s surface within these defined limits. For patients with a pre-pectoral positioned implant or those presenting with adverse factors such as pT3 disease, non-pathological complete response to widespread therapy, or invasion of the major pectoral muscle and/or chest wall, it is essential to incorporate the dorsal region between the implant and the pectoral muscle/chest wall into the clinical target volume (CTV). In our institution, the CTV in the ventral area of the implant was defined with an extra thickness of 3 mm to ensure comprehensive coverage of all subcutaneous lymphatic tissues and the pectoral muscle surrounding the implant. The planning target volume (PTV) was expanded by 3 mm to 5 mm from the CTV, with specific precautions to avoid the esophagus and lung. The PTV for the SCV should not extend medially toward the esophagus, while the PTV for the IMN must not encroach upon the lung. It may come into contact with the sternum but should not penetrate it. PMRT was administered using a hypofractionated schedule, delivering doses of 2.4 to 2.7 Gy (Gy) per fraction, resulting in a total radiation dose to the entire reconstructed chest wall ranging from 40.5 Gy to 45.9 Gy across 15 to 17 fractions. Regional nodal irradiation was delivered at a dosage comparable to that of the chest wall.

### Preoperative and postoperative assessments

**Fig. 1:**
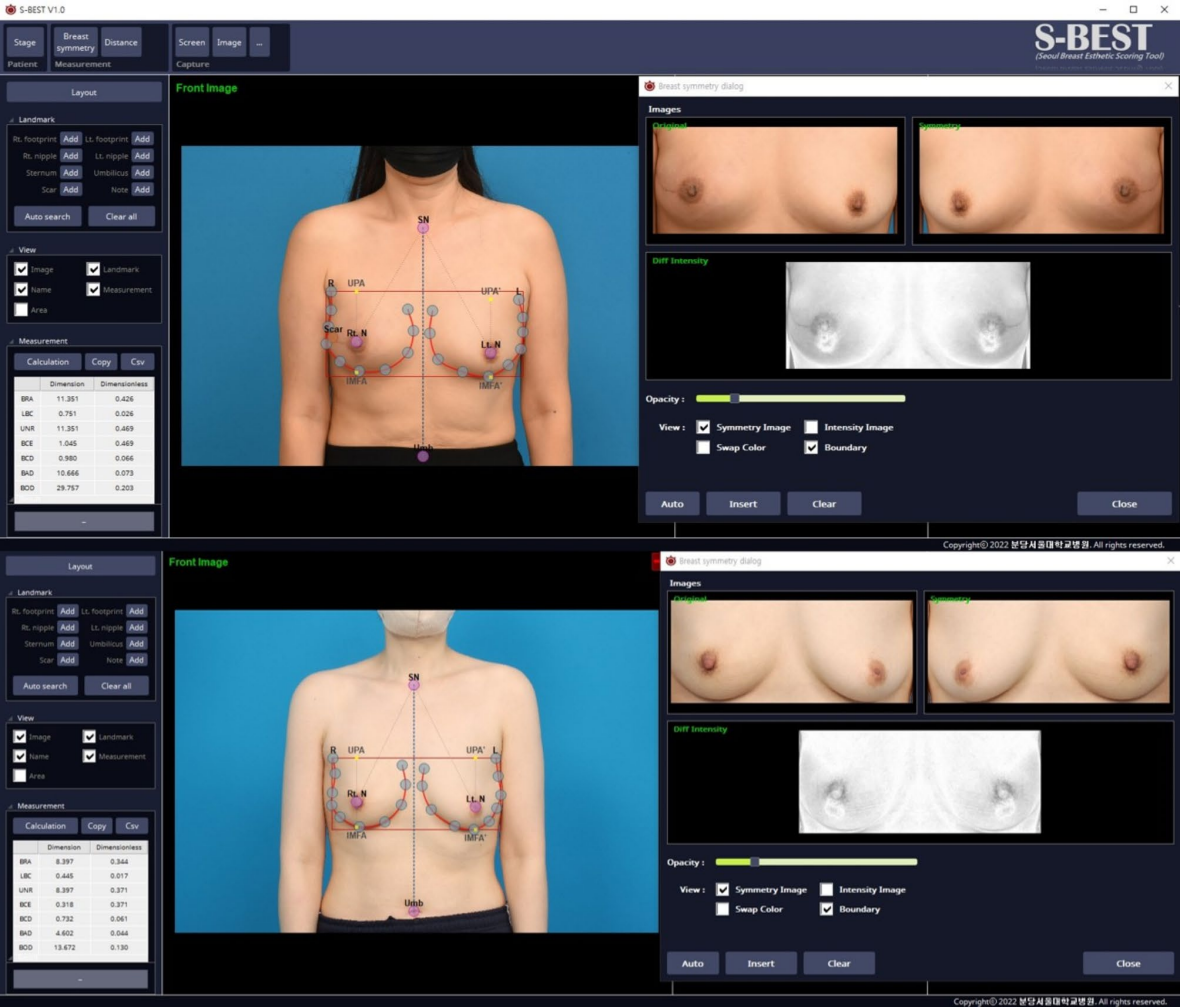
Screenshot of the S-BEST output page. Above) PR group. Below) IMF group. Landmarks are automatically detected using deep learning models. Breast asymmetry indices, including breast area difference (BAD), breast contour difference (BCD), breast compliance evaluation (BCE), breast overlap difference (BOD), and breast retraction assessment (BRA), are provided in the bottom left corner. On the right side of the screen, there is an image displaying an assessment of symmetry through the overlay of both breasts

Preoperative assessments and postoperative assessments (6 to 18 months post-surgery after PMRT sessions were completed) were completed for each participant. The total radiation dose received by each patient was noted in Gy units. The evaluations included detailed body measurements and medical photography, evaluated by the S-BEST software (Fig. 1).

The body measurements recorded were as follows: distance from the sternal notch to the nipple, distance from the nipple to the sternum, distance from the nipple to the IMF, and width of the breast base [12]. All photographs were taken by our in-hospital photographer using predetermined settings, including a camera-to-patient distance of 4 m, an aperture range of F/13–16, a 55–60 mm lens, ISO 200, along with a ceiling-mounted flash and a blue background. The photographs were captured from frontal, 45-degree, lateral, and side perspectives, with the arms positioned both raised and lowered throughout the session.

### S-BEST software

The S-BEST software (v1.0, 2022, Seoul, Republic of Korea), developed by our institution, was used for the objective evaluation of breast symmetry scores and the nipple Y-axis coefficient, which measures the distance from the clavicle to the nipple. Changes in the nipple-to-IMF distance were manually measured, with the average of three independent investigators’ measurements used to minimize error [12]. This deep-learning-based software, utilizing the DenseNet-264 model, automates breast landmark identification and calculates symmetry indices with high speed and accuracy. S-BEST detects 30 specific landmarks on frontal photographs, such as the sternal notch, nipples, and IMF, reducing the time and errors associated with manual evaluations. It provides both dimension-based metrics (e.g., distances in centimeters) and dimensionless indices (e.g., ratios), including breast area difference (BAD) and breast contour difference (BCD). Despite its strengths, S-BEST is limited to frontal photographs and cannot assess volumetric breast attributes.

### Statistical analysis

Descriptive statistics were used to summarize patient demographics and surgical details. The Shapiro-Wilk test was used to check the distribution of the dependent variables– aesthetic outcomes (breast symmetry scores, nipple-to-IMF distance changes, and nipple Y-axis coefficients) and the Kruskal-Wallis test was employed to compare the aesthetic outcomes across the three groups [13]. A multivariate linear regression analysis was conducted to identify predictors of aesthetic outcomes, including breast symmetry scores, nipple-to-IMF distance, and nipple Y-axis coefficients. Independent variables included incision type, radiation dose, age, and BMI. Receiver operating characteristic (ROC) curve analysis was performed to evaluate the predictive power of incision types and radiation dose on achieving optimal aesthetic outcomes (defined as symmetry scores within the top quartile). The area under the curve (AUC) was calculated to assess model performance. Additionally, the patients were divided into subgroups of cancer stage and pathology, and the Kruskal-Wallis test was employed to compare the aesthetic outcomes across each subgroup respectively. All statistical analyses were performed using SPSS software (version 25.0, IBM Corp., Armonk, NY, USA).

## Results

The demographics of the 40 patients (mean age: 45.85 years, range: 34–65), including BMI, average total radiation doses received (in Gy units), and the average changes in each parameter with corresponding p-values for group comparisons, are presented in Table 2. In this study, all participants reported no complications that greatly impacted the aesthetic results. The demographics of the PR and IMF groups were comparable, showing no significant differences in mean age (*p* = 0.67), BMI (*p* = 0.56), or radiation doses (*p* = 0.12). The Shapiro-Wilk test revealed that the breast symmetry score change followed a non-normal distribution (*p* < 0.001) while nipple-to-IMF distance change and nipple Y-axis coefficient change followed a normal distribution (*p* = 0.869, *p* = 0.392 respectively).

**Table 2:** Summary of patient demographics and aesthetic outcomes

	PR group	IMF group		*p*-value
Patients’ Number	9	31		
Average Age (years)	44.6	46.2		0.67
BMI (kg/m2)	23.9	23.6		0.56
Average Total Gray (Gy)	47.64 ± 5.2	54.23 ± 5.00		0.12
S-BEST outcomes		IMF1 (*n* = 13) (2019–2021)	IMF2 (*n* = 18) (2022)	
Breast symmetry score change (average)	-1.111 ± 0.78	-0.539 ± 0.78	-0.389 ± 0.92	0.11
Nipple to IMF distance change (cm, range)	0-0.2	0.2–0.5	0.3–0.4	0.17
Nipple y-axis coefficient change (average)	0.003 ± 0.060	-0.012 ± 0.041	-0.002 ± 0.040	> 0.05

BMI: Body mass index, PR: Periareolar/radial, IMF: Inframammary fold

The cancer stages and pathologies of the patients in this study are summarized in Table 3. In the PR group, there were three patients classified as Stage 0, three as Stage 1, one as Stage 2, and two as Stage 3. Within this group, three patients were diagnosed with DCIS, four with IDC, one with ILC, and one with MC. In the IMF group, the distribution of patients was as follows: 3 patients at Stage 0, 13 at Stage 1, 10 at Stage 2, and 5 at Stage 3. Four patients were diagnosed with DCIS, nineteen with IDC, four with a combination of IDC and ILC, and four with MC. These results demonstrate the distribution of cancer stages and histological subtypes between the two groups, indicating a more advanced cancer stage and a greater prevalence of IDC in the IMF group in comparison to the PR group.

**Table 3:** Summary of patients’ Cancer stages and pathologies

Group	Cancer Stage	Number of Patients	Pathology	Patients per Pathology
PR group	Stage 0	3	DCIS	3
	Stage 1	3	IDC	4
	Stage 2	1	ILC	1
	Stage 3	2	MC	1
IMF group	Stage 0	3	DCIS	4
	Stage 1	13	IDC	19
	Stage 2	10	IDC/ILC	4
	Stage 3	5	MC	4

PR: Periareolar/radial, IMF: Inframammary fold, DCIS: Ductal carcinoma in situ, IDC: Invasive ductal carcinoma, ILC: Invasive lobular carcinoma, MC: Mucinous carcinoma

Within the stage 1 group, there was a significant change in breast symmetry scores (*p* = 0.02) and nipple to IMF distance changes came close to significance (*p* = 0.057). Similarly, in stage 3, breast symmetry score change came close to significance (*p* = 0.059). However, the Kruskal-Wallis test revealed that there was no significant difference between all groups for any of the three aesthetic outcomes (Table 4). In pathology subgroup analysis, IDC group demonstrated a significant breast symmetry score change with *p* = 0.008 and IDC/ILC group exhibited changes in breast symmetry scores and nipple-to-IMF distance that approached significance (*p* = 0.059 and *p* = 0.063 respectively). Despite these findings, the Kruskal-Wallis test revealed that there is no significant difference between pathology subgroups for any aesthetic outcomes (Table 5).

**Table 4:** Cancer stage subgroup analysis results

Cancer Stage (number of patients)	Aesthetic Outcomes	Mean	*p*-value
0 (6)	Breast Symmetry Score changes	-0.5	0.18
	Nipple to IMF Distance changes	0.23	0.893
	Nipple Y-axis coefficient changes	-0.012	0.438
1(16)	Breast Symmetry Score changes	-0.56	0.02
	Nipple to IMF Distance changes	0.43	0.057
	Nipple Y-axis coefficient changes	0.012	0.156
2 (11)	Breast Symmetry Score changes	-0.45	0.129
	Nipple to IMF Distance changes	0.15	0.765
	Nipple Y-axis coefficient changes	0.0007	0.966
3 (7)	Breast Symmetry Score changes	-1	0.059
	Nipple to IMF Distance changes	-0.24	0.345
	Nipple Y-axis coefficient changes	0.0039	1
Differences among groups (Kruskal-Wallis test)		H-statistic	*p*-value
Breast Symmetry Score changes		1.58	0.6648
Nipple to IMF Distance changes		3.07	0.3805
Nipple Y-axis coefficient changes		2.35	0.5024

IMF: Inframammary fold

**Table 5:** Pathology subgroup analysis results

Pathology (number of patients)	Aesthetic Outcomes	Mean	*p*-value
MC (4)	Breast Symmetry Score changes	-0.25	0.564
	Nipple to IMF Distance changes	0.05	0.875
	Nipple Y-axis coefficient changes	0.033	0.109
ILC (1)	Breast Symmetry Score changes	0	N/A
	Nipple to IMF Distance changes	0	N/A
	Nipple Y-axis coefficient changes	0.009	N/A
IDC/ILC (5)	Breast Symmetry Score changes	-1	0.059
	Nipple to IMF Distance changes	0.8	0.063
	Nipple Y-axis coefficient changes	0.026	0.313
IDC (23)	Breast Symmetry Score changes	-0.61	0.008
	Nipple to IMF Distance changes	0.048	0.987
	Nipple Y-axis coefficient changes	-0.004	0.87
DCIS (7)	Breast Symmetry Score changes	-0.57	0.102
	Nipple to IMF Distance changes	0.41	0.463
	Nipple Y-axis coefficient changes	-0.004	0.688
Aesthetic outcome differences among groups		H-statistic	*p*-value
Breast Symmetry Score changes		2.261	0.668
Nipple to IMF Distance changes		3.586	0.465
Nipple Y-axis coefficient changes		3.979	0.409

MC: Mucinous carcinoma, ILC: Invasive lobular carcinoma, N/A: Not Applicable, IDC: Invasive ductal carcinoma, DCIS: Ductal carcinoma in situ, IMF: Inframammary fold

### Breast symmetry scores

**Fig. 2:**
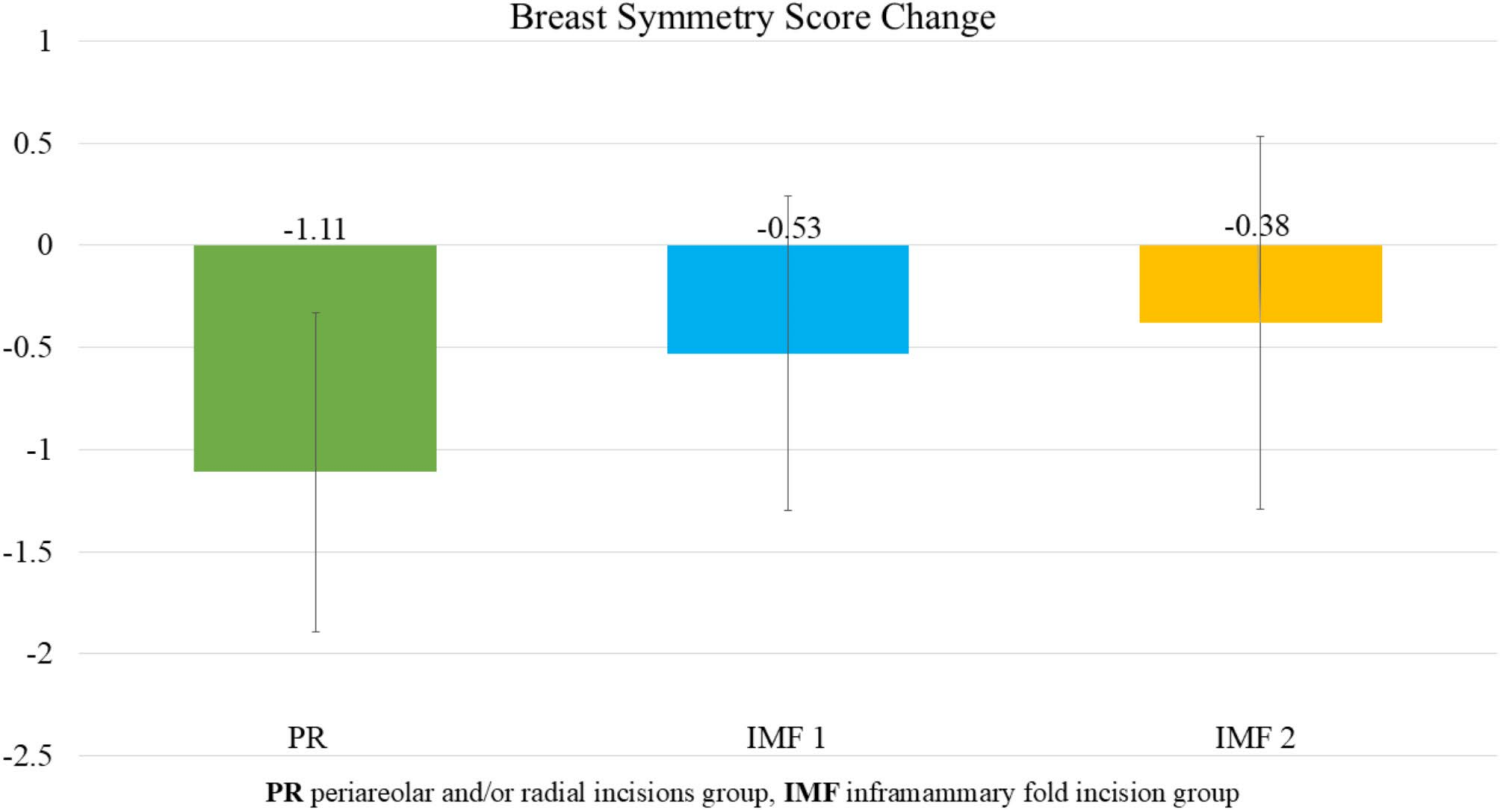
Average change in breast symmetry score among the three groups. All groups showed a decrease in breast symmetry scores postoperatively. The PR group had the most significant reduction, with a decrease of -1.111 ± 0.78. The IMF 1 group experienced a decrease of -0.539 ± 0.78, while the IMF 2 group showed the least reduction, with a decrease of -0.389 ± 0.92. These findings indicate that all incision types were associated with a decline in breast symmetry after NSM and PMRT, with the PR incision having the greatest impact. However, no statistically significant differences were observed between the groups

All groups showed a decrease in breast symmetry scores postoperatively. The PR group had the most significant reduction, with a decrease of -1.111 ± 0.78. The IMF 1 group experienced a decrease of -0.539 ± 0.78, while the IMF 2 group showed the least reduction, with a decrease of -0.389 ± 0.92. These findings, as presented in Table 6, indicate that all incision types were associated with a decline in breast symmetry after NSM and PMRT, with the PR incision having the greatest impact. However, no statistically significant differences were observed between the groups (*p* = 0.11; Fig. 2).

**Table 6:** Breast symmetry scores pre and post operation of all participants and average change of each group

Patients number	PR group		IMF 1 group			IMF 2 group			
	Pre	Post		Pre	Post		Pre	Post	
1	3	2		3	4		3	3	
2	2	2		3	2		4	3	
3	3	2		4	2		4	3	
4	3	2		3	3		4	4	
5	4	2		3	3		4	3	
6	3	3		4	3		3	3	
7	4	2		3	2		4	3	
8	4	2		3	3		4	2	
9	4	3		3	3		3	3	
10				3	2		4	4	
11				4	4		4	2	
12				3	2		2	2	
13				3	2		3	4	
14							3	4	
15							4	3	
16							4	3	
17							3	4	
18							3	3	
Average Change	-1.111 ± 0.78		-0.539 ± 0.78			-0.389 ± 0.92			
*p*-value	0.0027		0.0279			0.0896			

PR: Periareolar/radial, IMF inframammary fold, Pre: Preoperative symmetry scores,

Post: Postoperative symmetry scores

### Nipple-to-IMF distance

**Fig. 3:**
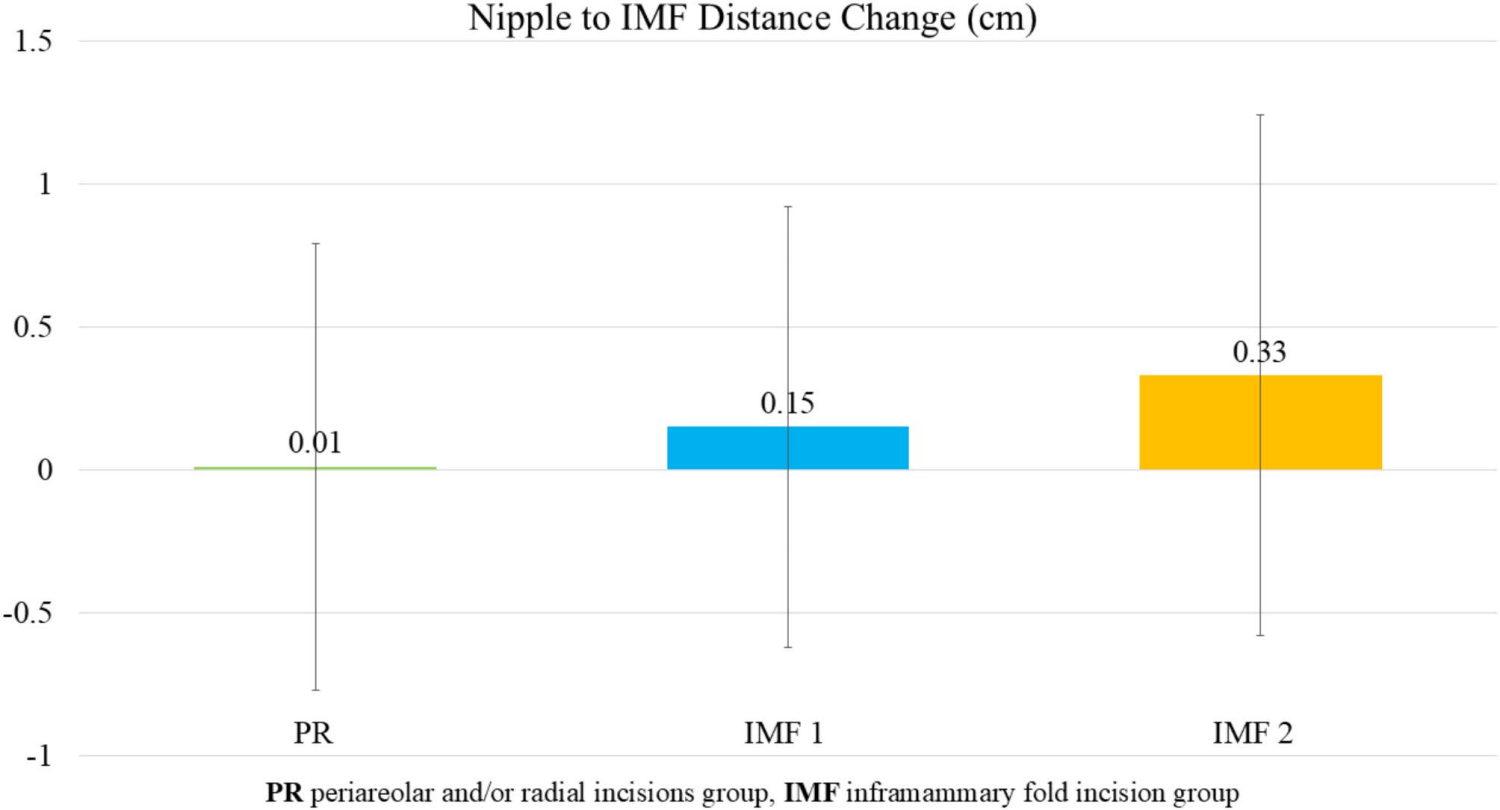
Average change in nipple-to-IMF distance among the three groups. An increase in the nipple-to-IMF distance was observed across all groups, reflecting changes in breast shape following radiation therapy. The PR group showed an increase ranging from 0 to 0.2 cm, the IMF 1 group experienced an increase of 0.2 to 0.5 cm, and the IMF 2 group had an increase of 0.3 to 0.4 cm. This increase suggests a potential radiation-induced effect on breast tissue, with the PR group showing the smallest increment. However, the differences between groups were not statistically significant (*p* = 0.17)

An increase in the nipple-to-IMF distance was observed across all groups, reflecting changes in breast shape following radiation therapy. The PR group showed an increase ranging from 0 to 0.2 cm, the IMF 1 group experienced an increase of 0.2 to 0.5 cm, and the IMF 2 group had an increase of 0.3 to 0.4 cm, as detailed in Table 7. This increase suggests a potential radiation-induced effect on breast tissue, with the PR group showing the smallest increment. However, the differences between groups were not statistically significant (*p* = 0.17), as illustrated in Fig. 3.

**Table 7:** Nipple to IMF distance in cm for pre and post operation of all participants and average change of each group

Patients Number	PR group			IMF 1 group		IMF 2 group	
	Pre (cm)		Post(cm)	Pre(cm)	Post(cm)	Pre(cm)	Post(cm)
1	6.0	7.1		5	5.3	3	3.4
2	4.2	4.8		4.1	5.3	5.5	5.2
3	6.9	6.7		7.6	6.7	5.2	6.7
4	4.9	4.3		4.9	4.8	4.2	4
5	6.8	5.6		4.3	4.8	5.2	7.3
6	4.2	4.2		3.1	3.2	2.5	3.9
7	4	4.7		5.4	4.8	2.5	4.2
8	5.5	5.5		4.9	3.4	4.8	3.9
9	4.3	4.1		4.8	4.9	6.9	5.3
10				4.9	4.4	3.4	4.4
11				4.7	5.5	4.4	3.6
12				4	5.6	3.8	4.2
13				4.7	5.7	3.1	5.6
14						2.3	1.8
15						3.3	2.1
16						2.8	3.6
17						3	3
18						4.7	4.4
Average Change	0.01 ± 0.70			0.15 ± 0.88		0.33 ± 1.18	
*p*-value	0.9266			0.5420		0.2469	

PR: Periareolar/radial, IMF: Inframammary fold, Pre: Preoperative symmetry distances,

Post: Postoperative symmetry distances

### Nipple Y-axis coefficient change

**Fig. 4:**
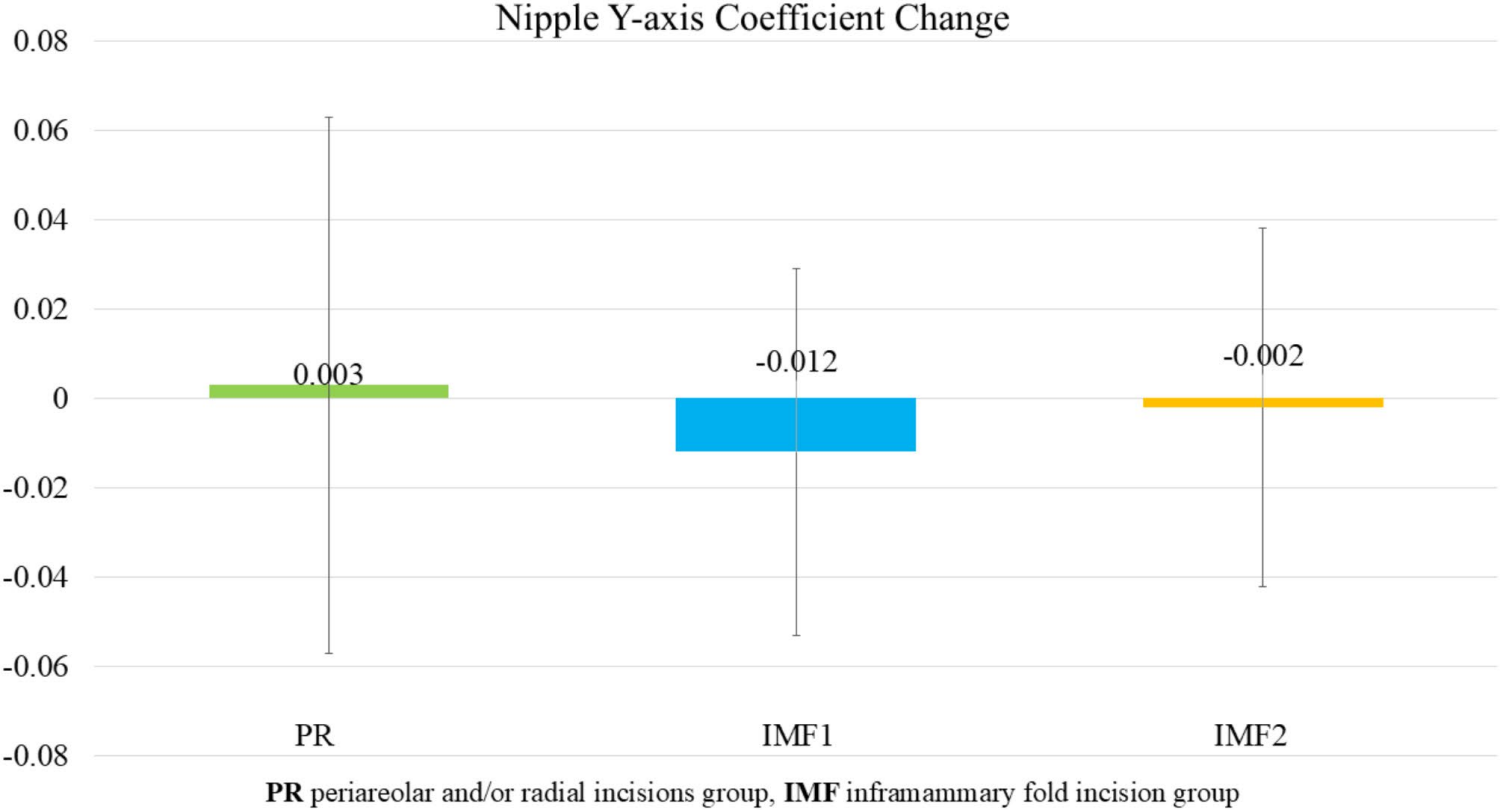
Average change in nipple Y-axis level among the three groups. All groups demonstrated a slight increase in the nipple Y-axis level after radiation therapy, with changes remaining below 0.012. No statistically significant differences were observed between the groups (*p* > 0.05)

All groups demonstrated a slight increase in the nipple Y-axis level after radiation therapy, with changes remaining below 0.012, as shown in Table 8. No statistically significant differences were observed between the groups (*p* > 0.05), as illustrated in Fig. 4.

**Table 8:** Nipple Y-axis coefficient pre and post operation of all participants and average change of each group

Patients Number	PR group			IMF 1 group			IMF 2 group	
	Pre	Post	Pre		Post	Pre		Post
1	0.384	0.465	0.474		0.557	0.348		0.434
2	0.348	0.432	0.493		0.477	0.406		0.418
3	0.326	0.318	0.438		0.411	0.405		0.445
4	0.356	0.307	0.37		0.359	0.35		0.386
5	0.46	0.428	0.421		0.367	0.445		0.44
6	0.425	0.434	0.412		0.432	0.379		0.378
7	0.391	0.44	0.358		0.37	0.356		0.356
8	0.395	0.302	0.303		0.339	0.345		0.316
9	0.504	0.436	0.44		0.413	0.547		0.496
10			0.379		0.36	0.374		0.408
11			0.349		0.379	0.388		0.275
12			0.26		0.341	0.425		0.423
13			0.475		0.517	0.378		0.392
14						0.396		0.386
15						0.453		0.472
16						0.417		0.42
17						0.385		0.373
18						0.425		0.433
Average Change	0.003 ± 0.060			-0.012 ± 0.041			-0.002 ± 0.040	
*p*-value	0.8917			0.3433			0.8697	

PR: Periareolar/radial, IMF: Inframammary fold, Pre: Preoperative symmetry coefficient,

Post: Postoperative symmetry coefficient

### Comparison of aesthetic outcomes across incision types

As the study involved small sample size and the Shapiro-Wilk test results revealed non-normal distribution for breast symmetry score change, the Kruskal-Wallis test was used to evaluate differences in breast symmetry scores, nipple-to-IMF distance, and nipple Y-axis coefficient changes among the three groups. The analysis revealed no significant differences in these metrics (*p* > 0.05), indicating that the incision type, whether PR or IMF incisions, does not have a significant impact on aesthetic outcomes.

### Multivariate regression analysis

The multivariate regression analysis did not identify any statistically significant predictors of breast symmetry score change, nipple to IMF distance change, or nipple Y-axis coefficient change (*p* > 0.05) as shown in Table 9. However, radiation dose approached statistical significance with a p-value of 0.074 was observed for nipple to IMF distance change, indicating a potential association. Nevertheless, none of the independent variables - incision type (*p* = 0.092, β = 0.306), age (*p* = 0.666, β = -0.071), BMI (*p* = 0.814, β = -0.040), and radiation dose (*p* = 0.944, β = 0.013) - significantly influenced postoperative symmetry.

**Table 9:** Multivariate regression analysis results

Dependent Variable	Independent Variables	*p*-value	Beta (β)	95% Confidence Interval	
				Min.	Max.
Breast Symmetry Change	Incision Type	0.092	0.306	-0.059	0.744
	Age	0.666	-0.071	-0.056	0.036
	BMI	0.814	-0.040	-0.136	0.107
	Radiation Dose	0.944	0.013	-0.053	0.057
Nipple to IMF Distance Change	Incision Type	0.211	0.219	-0.164	0.717
	Age	0.220	-0.198	-0.082	0.020
	BMI	0.416	0.135	-0.079	0.187
	Radiation Dose	0.074	-0.319	-0.115	0.006
Nipple Y-axis Coefficient	Incision Type	0.864	-0.032	-0.023	0.020
	Age	0.422	-0.138	-0.003	0.001
	BMI	0.824	-0.039	-0.007	0.006
	Radiation Dose	0.914	-0.020	-0.003	0.003

BMI: Body mass index, IMF: Inframammary fold

### ROC curve analysis

**Fig. 5:**
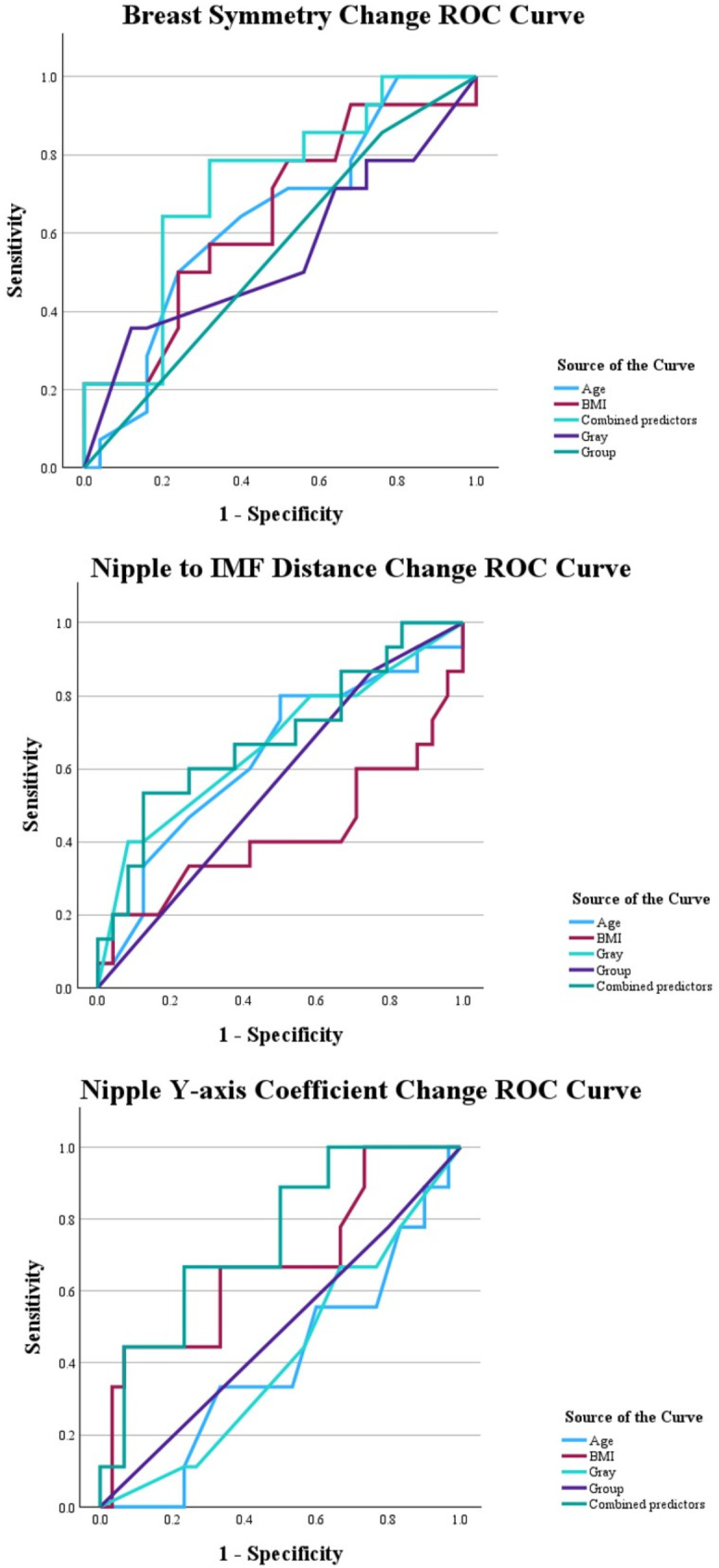
ROC curves for each dependent variable– (**A**) Breast symmetry change, (**B**) Nipple to IMF distance change, and (**C**) Nipple Y-axis coefficient change. The AUC of combined predictors for A is 0.723, B 0.686, and C 0.744. The best cut-off points for A are group (0.760, 0.857) with Youden’s index 0.097, age (0.240, 0.500) with 0.260, BMI (0.520, 0.786) with 0.266, radiation dose (0.120, 0.357) with 0.237, and combined predictors (0.320, 0.786) with 0.466. The best cut-off points for B are group (0.750, 0.867) with Youden’s index 0.117, age (0.500, 0.800) with 0.300, BMI (0.083, 0.200) with 0.117, radiation dose (0.083, 0.400) with 0.317, and combined predictors (0.125, 0.533) with 0.408. Finally, the best cut-off points for C are group (0.800, 0.778) with Youden’s index − 0.220, age (0.967, 1.00) with 0.033, BMI (0.100, 0.444) with 0.344, radiation dose (0.667, 0.667) with 0, and combined predictors (0.233, 0.667) with 0.433

The ROC curve analysis revealed that combined predictors (incision type, age, BMI, and radiation dose) exhibited a moderate predictive value for achieving optimal symmetry outcomes, defined as no change in breast symmetry scores, with an AUC of 0.72 (95% CI: 0.56–0.89)(Fig. 5). Similarly, the predictive value of combined predictors for optimal nipple Y-axis coefficient change, defined as change of less than 0.01, yielded an AUC of 0.74 (95% CI: 0.57–0.92). The best cut-off points for breast symmetry change curves are group (0.760, 0.857) with Youden’s index 0.097, age (0.240, 0.500) with 0.260, BMI (0.520, 0.786) with 0.266, radiation dose (0.120, 0.357) with 0.237, and combined predictors (0.320, 0.786) with 0.466. The best cut-off points for nipple to IMF distance change curves are group (0.750, 0.867) with Youden’s index 0.117, age (0.500, 0.800) with 0.300, BMI (0.083, 0.200) with 0.117, radiation dose (0.083, 0.400) with 0.317, and combined predictors (0.125, 0.533) with 0.408. Finally, the best cut-off points for nipple Y-axis coefficient curves are group (0.800, 0.778) with Youden’s index − 0.220, age (0.967, 1.00) with 0.033, BMI (0.100, 0.444) with 0.344, radiation dose (0.667, 0.667) with 0, and combined predictors (0.233, 0.667) with 0.433.

## Discussion

The findings of this study demonstrate that both PR and IMF incisions lead to observable changes in aesthetic outcomes following NSM with prepectoral DTI reconstruction and subsequent PMRT. Although all groups showed decreases in breast symmetry scores and increases in nipple-to-IMF distance, the differences between the groups were not statistically significant. This suggests that, at least from a quantitative perspective, the choice of incision type may not significantly influence the aesthetic outcomes in terms of breast symmetry and breast shape alterations. However, specific trends observed in the data suggest practical considerations regarding incision selection.

The PR incision group showed the most pronounced decrease in breast symmetry scores. This may be partly due to the location and orientation of the incision, which could differently affect tissue exposure to radiation and subsequent healing processes. PR incisions, positioned around the NAC, has been linked to increased risk of NAC necrosis and heightened surgical complications in some studies [7, 14, 15]. This suggests that the PR incision, while advantageous in certain surgical contexts, may require additional caution, especially for patients anticipated to undergo radiation therapy [16].

In contrast, the IMF incision group showed better symmetry scores, consistent with existing literature associating IMF incisions with less visible scarring and more favorable cosmetic outcomes [17]. These aesthetic advantages may be attributed to the IMF incision’s less conspicuous location, which allows for better scar concealment, and the incision distance from the NAC, potentially reducing the risk of tissue damage in the sensitive area [18]. Although this study did not find statistically significant differences, the symmetry-related benefits observed with IMF incisions suggest that the choice of incision type may have subtle but meaningful effects on aesthetic outcomes.

Asymmetry following breast cancer surgery, particularly after NSM with reconstruction, is often influenced by molecular and cellular responses to surgical and radiation-induced trauma. One of the primary factors is radiation-induced fibrosis, characterized by an excessive production and deposition of extracellular matrix (ECM) proteins such as collagen. This fibrosis results from the activation of fibroblasts into myofibroblasts, driven by cytokines like transforming growth factor-beta (TGF-β). Elevated TGF-β levels promote tissue stiffening, contracture, and scarring, leading to visible asymmetry in breast contour and positioning [19].

Another contributor is vascular endothelial damage, which impairs tissue perfusion and healing. Radiation therapy induces oxidative stress, damaging endothelial cells and reducing capillary density. This can result in uneven tissue elasticity and volume loss, exacerbating asymmetry. Additionally, adipocyte dysfunction in the irradiated breast tissue, mediated by pro-inflammatory cytokines such as interleukin-6 (IL-6) and tumor necrosis factor-alpha (TNF-α), can lead to fat necrosis and volume discrepancies. The cellular response to hypoxia also plays a critical role. Hypoxia-inducible factors (HIFs) are upregulated in poorly perfused tissue, triggering angiogenesis and fibrosis. While these adaptive mechanisms aim to restore oxygenation, they can lead to uneven tissue remodeling, further contributing to asymmetry.

Finally, differences in stem cell activity in adipose-derived stromal cells (ADSCs) between irradiated and non-irradiated tissues may influence tissue regeneration and symmetry. Understanding these molecular and cellular mechanisms highlights potential therapeutic targets, such as antifibrotic agents or regenerative therapies, to mitigate asymmetry and improve reconstructive outcomes in breast cancer surgery.

An analysis of total radiation doses adds further depth to these findings. The PR group received a slightly lower average radiation dose (47.64 ± 5.2 Gy) than both IMF groups (IMF1 group: 54.45 ± 5.28 Gy, IMF2 group: 54.07 ± 4.79 Gy). Despite higher doses, the IMF incisions appeared to maintain greater aesthetic stability, suggesting that IMF incisions may better withstand more severe radiation induced changes in symmetry and scar visibility [20, 21]. Previous studies have indicated that tissue near the inframammary fold may experience less radiation associated fibrosis, protecting against severe scarring and contraction effects compared to tissues closer to the NAC [22–24]. These findings align with literature suggesting that IMF incisions, due to their hidden location and relative distance from critical vascular areas, may have a structural advantage in maintaining cosmetic outcomes even under higher radiation doses [20, 21]​.

Previous literature comparing incision types has predominantly focused on complications rather than aesthetic outcomes. For instance, Salibian et al. compared IMF incisions and lateral radial incisions in NSM with immediate microvascular breast reconstruction and found that IMF incisions were associated with higher rates of major ischemic complications compared to lateral radial incisions [25]. Similarly, another study reported that the breakdown of the total skin-sparing mastectomy incision was twice as likely in the IMF group compared to other incision types [26]. Conversely, Frey et al. highlighted that IMF incisions were the most protective of overall complications, with vertical radial incisions ranking next [27]. Other studies reported no significant differences between incision types [17, 28, 29].

In the cancer stage subgroup analysis, while stage 1 patients demonstrated a significant change in breast symmetry scores (*p* = 0.02), the changes in nipple-to-IMF distance only approached statistical significance (*p* = 0.057). Similarly, in stage 3, breast symmetry score changes neared significance (*p* = 0.059). Despite these findings, the Kruskal-Wallis test indicated no significant differences in aesthetic outcomes across all groups, suggesting that overall stage classification may not be a primary determinant of post-treatment symmetry and proportion. Further analysis based on pathology showed that the IDC group exhibited a statistically significant change in breast symmetry scores (*p* = 0.008). Additionally, in the IDC/ILC group, changes in breast symmetry scores (*p* = 0.059) and nipple-to-IMF distance (*p* = 0.063) approached significance. These results imply that specific pathological characteristics may contribute to variations in aesthetic outcomes, potentially due to differences in tumor biology, surgical techniques, or healing responses. However, the lack of a significant difference among groups in the Kruskal-Wallis test suggests that these changes may be subtle and influenced by multiple confounding factors.

The absence of statistically significant differences between groups in the Kruskal-Wallis test may be attributed to sample size limitations, inter-patient variability, or the complex interplay of surgical and physiological factors affecting aesthetic outcomes. Additionally, while individual comparisons within groups revealed meaningful trends, these findings should be interpreted with caution due to the multiple comparisons made. Future studies with larger sample sizes and standardized aesthetic evaluation criteria are warranted to further clarify the impact of clinical stage and pathology on breast symmetry and proportion.

Several complications can influence the cosmetic results of the procedure. A notable complication arising from radiotherapy is capsular contracture, alongside additional potential issues including seroma, hematoma, flap necrosis, and infection, all of which can adversely affect aesthetic outcomes. Seromas and hematomas can contribute to delayed capsular contracture, whereas flap necrosis may cause important deformities, including displacement of the NAC. Infections that contribute to capsular contracture can considerably impact aesthetic outcomes. Besides, when implant removal is necessary, obtaining satisfactory cosmetic results during reimplantation may prove to be difficult.

A limited number of studies have specifically addressed the aesthetic outcomes associated with different incision types. One study noted that hidden incisions, such as IMF and scarless periareolar incisions; or vertical incisions like radial incisions, scored the highest for self-reported aesthetic satisfaction compared to transverse incisions (e.g. lateral, circum-lateral, and transverse) [26]. Another compared hemi-periareolar, periareolar, vertical, and wise incisions and found no significant difference in self-reported satisfaction of the aesthetic results [30]. The lack of focused comparisons on aesthetic results with objective parameters underscores the importance of this study.

While this study did not identify statistically significant multivariate regression analysis, the trends observed warrant further investigation. The correlations identified between radiation dosage and changes in the distance from the nipple to the inframammary fold indicate that this factor may have an impact on the outcomes. Lower radiation dose seems to correlate with enhanced aesthetic results. Given this trend, it is essential to conduct further research to assess the predictive significance of radiation dosage and to investigate the influence of these two factors more comprehensively with larger sample sizes.

Additionally, while incision type, age, BMI, and radiation dose did not significantly influence aesthetic outcomes, the variables combined exhibited moderate predictive value in the ROC curve analysis, highlighting the potential importance of patient-specific factors, such as tissue quality and surgical technique, in determining aesthetic results. The combined predictors achieved AUC values of 0.72 and 0.74 for optimal breast symmetry score change and nipple Y-axis coefficient changes respectively, suggesting that these factors may collectively influence postoperative aesthetic results, reinforcing the importance of comprehensive preoperative planning. Moving forward, efforts to minimize radiation dose, where clinically appropriate, may enhance aesthetic outcomes. The results indicate that a larger sample size could reveal that these variables greatly influence postoperative outcomes, emphasizing the necessity for customized treatment strategies. Moreover, further exploration of patient-specific factors, such as tissue elasticity, wound healing capacity, and surgical precision may provide deeper insights into optimizing the aesthetic outcomes.

From a clinical perspective, these findings suggest that the choice between IMF and PR incisions should be guided not solely by anticipated aesthetic outcomes but also by patient specific factors such as anatomy, personal preference, and the potential visibility of scars, rather than significant differences in post-radiation aesthetic outcomes [31]. The potential preference for IMF incision due to less visible scarring offers an important consideration for surgical planning [17]. However, this choice must also take into consideration the oncologic safety and patient priorities, balancing long-term aesthetic considerations with safety and effective cancer control [32].

### Limitations

This study has several limitations that should be considered when interpreting the results. First of all, the relatively small sample size, particularly in the PR group, limits the statistical power to detect subtle differences between groups. In the study design, a retrospective methodology was used, focusing on a defined time frame and selecting patients who fulfilled the criteria. This led to variations in sample sizes between the two groups. The choice of incision was not arbitrary, nor was there a deliberate preference for a particular type of incision. The decision was made considering the patient’s condition and the surgeon’s clinical assessment. As a result, it was impractical to enlarge the sample size of one group by increasing the number of surgeries with a specific incision, which posed a major challenge in addressing this limitation. In light of these challenges, we employed a strong study design, measurable outcomes, and a comprehensive evaluation of potential issues to perform an accurate comparative analysis between the groups. In addition, the information pertaining to each specific participant involved in the study can be found detailed in Tables. These tables provide a comprehensive overview of the data collected for every individual subject included in the research.

Second, while objective tools such as the S-BEST software were employed to assess aesthetic outcomes, the subjective nature of visual assessments may still introduce bias [33]. The measurement of distances between landmarks is based on a two-dimensional image, which may result in variations when compared to the true distances. This observation is especially relevant when assessing the distance from the nipple to IMF, as the distance between these landmarks, when viewed from the front, is often perceived as being shorter than it truly is.

Third, the study did not include patient-reported outcome measures, which are essential for capturing subjective satisfaction and quality of life. Additionally, the follow-up period of 6 to 18 months may not fully account for long-term changes in aesthetic outcomes, such as those arising from progressive radiation-induced fibrosis or implant-related complications. Finally, the study was conducted at a single institution, which may limit the generalizability of the findings to other patient populations or surgical practices.

Future research should address these limitations by incorporating larger, multicenter prospective studies with longer follow-up periods and including both objective and subjective outcome measures to provide a more comprehensive understanding of the factors influencing aesthetic outcomes in NSM and reconstruction. Additionally, incorporating patient demographics, such as breast size, could enable more tailored recommendations for incision types, ensuring that surgical planning aligns with both oncologic safety and individual patient preferences. Finally, incorporating patient-reported outcomes will be critical to holistically capture satisfaction and quality of life, bridging the gap between clinical measures and patient-centered priorities.

## Conclusion

Our study demonstrates that while both IMF and PR incisions for NSM followed by prepectoral DTI reconstruction and PMRT are associated with changes in aesthetic outcomes, there is no significant differences between the two incision types in terms of breast and nipple symmetry. However, the observed trends suggest that IMF incisions may offer better aesthetic results, particularly for patients anticipated to receive higher radiation doses. Continued research is needed to explore the complex interplay between surgical techniques, radiation therapy, and aesthetic outcomes to optimize the care for breast cancer patients undergoing mastectomy and reconstruction.

## Electronic supplementary material

Below is the link to the electronic supplementary material.

Supplementary Material 1

## Data Availability

Data is provided within the manuscript.

## Abbreviations

IMF: Inframammary fold
PR: Periareolar/radial (radial incision with or without a periareolar incision)
NSM: Nipple-sparing mastectomy
DTI: Direct-to-implant
PMRT: Post-mastectomy radiotherapy
S-BEST: Seoul breast esthetic scoring tool
NAC: Nipple areolar complex
RT: Radiotherapy
BMI: Body mass index
ADM: Acellular dermal matrix
IMN: Internal mammary nodes
SCV: Supraclavicular region
CTV: Clinical target volume
PTV: Planning target volume
Gy: Gray
BAD: Breast area difference
BCD: Breast contour difference
ROC: Receiver operating characteristic
AUC: Area under the curve
DCIS: Ductal carcinoma in situ
IDC: Invasive ductal carcinoma
ILC: Invasive lobular carcinoma
MC: Mucinous carcinoma
ECM: Extracellular matrix
TGF-β: Transforming growth factor-beta
IL-6: Interleukin-6
TNF- α: Tumor necrosis factor-alpha
HIFs: Hypoxia-inducible factors
ADSCs: Adipose-derived stromal cells
BCE: Breast compliance evaluation
BOD: Breast overlap difference
BRA: Breast retraction assessment
