# Supplementary material for: Assessing aesthetic outcomes of different incision types for nipple-sparing mastectomy followed by radiation therapy in prepectoral direct-to-implant breast reconstruction: a retrospective study
Source: World J Surg Oncol. 2025 Mar 15;23:91. doi: 10.1186/s12957-025-03730-4 (PMC11909819; doi:10.1186/s12957-025-03730-4)
Supplement: Supplementary file 1 — Supplementary Material 1 [file 12957_2025_3730_MOESM1_ESM.docx]

STROBE Statement—Checklist of items that should be included in reports of observational studies

|  | | Item No | Recommendation |
| --- | --- | --- | --- |
| **Title and abstract** | | 1 | (*a*) Indicate the study’s design with a commonly used term in the title or the abstract  Applied Retrospective study as stated in the title and Patients and Methods in abstract on pages 1,3 and 4 |
|  |  |  | (*b*) Provide in the abstract an informative and balanced summary of what was done and what was found  Provided in Abstract on pages 3 and 4. |
| Introduction | | | |
| Background/rationale | | 2 | Explain the scientific background and rationale for the investigation being reported  Included in the Introduction on page 5. |
| Objectives | | 3 | State specific objectives, including any prespecified hypotheses  Included in the Introduction on page 6. |
| Methods | | | |
| Study design | | 4 | Present key elements of study design early in the paper  Included in the Patients on page 7. |
| Setting | | 5 | Describe the setting, locations, and relevant dates, including periods of recruitment, exposure, follow-up, and data collection  Included in the Patients on page 7. |
| Participants | | 6 | (*a*) Give the eligibility criteria, and the sources and methods of selection of participants. Describe methods of follow-up  Included in the Patients and Samples on pages 7, 8 and Table 1. |
|  |  |  | (*b*) *Cohort study*—For matched studies, give matching criteria and number of exposed and unexposed  Not applicable. |
| Variables | | 7 | Clearly define all outcomes, exposures, predictors, potential confounders, and effect modifiers. Give diagnostic criteria, if applicable  Included in the Patients on pages 7-11. |
| Data sources/ measurement | | 8* | For each variable of interest, give sources of data and details of methods of assessment (measurement). Describe comparability of assessment methods if there is more than one group  Included in the Patients on pages 9 and 10. |
| Bias | | 9 | Describe any efforts to address potential sources of bias  Addressed in applying strict inclusion and exclusion criteria on page 7, using standardized assessments (S-BEST section on page 10), performing statistical analysis for confounders, and ensuring uniform clinical protocols within a single institution. |
| Study size | | 10 | Explain how the study size was arrived at  Included in the Methods on page 8. |
| Quantitative variables | | 11 | Explain how quantitative variables were handled in the analyses. If applicable, describe which groupings were chosen and why  Included in the Patients on pages 9-11. |
| Statistical methods | | 12 | (*a*) Describe all statistical methods, including those used to control for confounding  Included in Statistical analysis on pages 10 and 11. |
|  |  |  | (*b*) Describe any methods used to examine subgroups and interactions  Included in the Statistical analysis on page 11. |
|  |  |  | (*c*) Explain how missing data were addressed  Included in the Patients exclusion criteria on pages 7 and 8. Missing data were addressed by excluding from the study, ensuring that only complete and reliable data were analysed. |
|  |  |  | (*d*) If applicable, explain how loss to follow-up was addressed  Included in the Patients exclusion criteria on pages 7 and 8. |
|  |  |  | (*e*) Describe any sensitivity analyses  Included in the page 11. |
| Results | | | |
| Participants | 13* | (a) Report numbers of individuals at each stage of study—eg numbers potentially eligible, examined for eligibility, confirmed eligible, included in the study, completing follow-up, and analysed  Included in the Results on page 12. | |
|  |  | (b) Give reasons for non-participation at each stage  Included in the page 7. Exclusion criteria | |
|  |  | (c) Consider use of a flow diagram  Included in the page 12. Table 2 | |
| Descriptive data | 14* | (a) Give characteristics of study participants (eg demographic, clinical, social) and information on exposures and potential confounders  Included in the Results on page 12. | |
|  |  | (b) Indicate number of participants with missing data for each variable of interest  Included in the page 7. Exclusion criteria | |
|  |  | (c) Summarise follow-up time (eg, average and total amount)  Included in the Results . | |
| Outcome data | 15* | Report numbers of outcome events or summary measures over time  Included in the page 12. Table 2. | |
| Main results | 16 | (*a*) Give unadjusted estimates and, if applicable, confounder-adjusted estimates and their precision (eg, 95% confidence interval). Make clear which confounders were adjusted for and why they were included  Included in the pages 12-14. Tables 2-6 | |
|  |  | (*b*) Report category boundaries when continuous variables were categorized  Included in the page 7, and summarized in Tables 1 and 2 | |
|  |  | (*c*) If relevant, consider translating estimates of relative risk into absolute risk for a meaningful time period.  Not applicable. | |
| Other analyses | 17 | Report other analyses done—eg analyses of subgroups and interactions, and sensitivity analyses  Included in the pages 12-14 (Regression and ROC Curve Analysis) on pages 14 and 15 | |
| Discussion | | | |
| Key results | 18 | Summarise key results with reference to study objectives  Included in the page 15. | |
| Limitations | 19 | Discuss limitations of the study, taking into account sources of potential bias or imprecision. Discuss both direction and magnitude of any potential bias  Included in the Discussion limitation section on pages 20 and 21. | |
| Interpretation | 20 | Give a cautious overall interpretation of results considering objectives, limitations, multiplicity of analyses, results from similar studies, and other relevant evidence  Included in the Discussion on pages 18 and 19. | |
| Generalisability | 21 | Discuss the generalisability (external validity) of the study results  Included in the Discussion on page 16-19. | |
| Other information | | | |
| Funding | 22 | Give the source of funding and the role of the funders for the present study and, if applicable, for the original study on which the present article is based  Funding provided in the text on pages 24. BSP 20240603. | |
